# Supplementary material for: Safety and Immunogenicity of the Convacell® Recombinant N Protein COVID-19 Vaccine
Source: Vaccines (Basel). 2024 Jan 19;12(1):100. doi: 10.3390/vaccines12010100 (PMC10821050; doi:10.3390/vaccines12010100)
Supplement: Supplementary file 1 [file vaccines-12-00100-s001.zip › vaccines-2785632-supplementary.pdf]

Safety and Immunogenicity of the Convacell® Recombinant N Protein COVID-19 Vaccine

Sevastyan O. Rabdano[1*], Ellina A. Ruzanova[1], Denis S. Makarov[1], Anastasiya E. Vertyachikh[1], Valeriya A. Teplykh[1], German O. Rudakov[1], Iuliia. V. Pletyukhina[1], Nikita S. Saveliev[1], Konstantin A. Zakharov[2], Diana N. Alpenidze[3], Vasiliy B. Vasilyuk[4], Sergei A. Arakelov[1] and Veronika I. Skvortsova[5]

[1] Saint Petersburg Scientific Research Institute of Vaccines and Serums of the Federal Medical-Biological Agency of Russia (SPbSRIVS), St. Petersburg, Russia, 198320

[2] LLC "NIC Eco-bezopasnost", St. Petersburg, Russia, 191119

[3] State Budgetary Health Institution "City Polyclinic No. 117", St. Petersburg, Russia, 194358

[4] Department of Toxicology, Extreme and Diving Medicine, North-Western State Medical University named after I.I. Mechnikov: St. Petersburg, Russia, 191015

[5] Federal Medical-Biological Agency of Russia, Moscow, Russia, 115682

* Correspondence: sevastyan@rabdano.ru

Table S1: Volunteer demographic data overview for phase I.

Group	Variable	Age (full years)	Weight (kg)	Height (cm)	BMI (kg/m^2)
1	n	5	5	5	5
	M	26.00	67.30	172.00	22.70
	SI	9.03	9.28	9.14	1.87
	95% CI	14.79 – 37.21	55.77 – 78.83	160.65 – 183.35	20.38 – 25.02
	range	21.00 – 42.00	60.00 – 82.00	164.00 – 186.00	19.85 – 24.86
	Me	22.00	62.00	169.00	22.77
	Q1 – Q3	21.00 – 24.00	61.50 – 71.00	165.00 – 176.00	22.31 – 23.70
2	n	15	15	15	15
	M	27.00	66.25	170.73	22.69
	SI	5.89	11.41	8.85	3.37
	95% CI	23.74 – 30.26	59.93 – 72.57	165.83 – 175.64	20.83 – 24.56
	range	19.00 – 37.00	50.60 – 86.50	158.00 – 193.00	18.59 – 29.93
	Me	25.00	64.00	170.00	21.38
	Q1 – Q3	22.50 – 32.00	59.85 – 70.00	166.00 – 173.50	20.34 – 24.08

Table S2: Volunteer demographic data overview for phase II.

Group	Variable	Age (full years)	Weight (kg)	Height (cm)	BMI (kg/m^2)
3	n	44	44	44	44
	M	28.34	67.17	171.98	22.66
	SI	7.07	10.10	10.13	2.40
	95% CI	26.19 – 30.49	64.10 – 70.24	168.90 – 175.06	21.94 – 23.39
	range	18.00 – 50.00	52.10 – 98.00	151.00 – 191.00	19.44 – 29.76
	Me	27.00	67.00	172.00	22.62
	Q1 – Q3	23.00 – 31.25	59.52 – 74.42	165.75 – 180.00	20.62 – 23.89
4	n	45	45	45	45
	M	29.80	73.11	176.20	23.52
	SI	9.62	10.85	9.21	2.73
	95% CI	26.91 – 32.69	69.85 – 76.37	173.43 – 178.97	22.70 – 24.34
	range	18.00 – 60.00	54.80 – 100.00	153.00 – 197.00	18.52 – 29.07
	Me	27.00	74.40	175.00	23.55
	Q1 – Q3	23.00 – 33.00	63.70 – 80.80	170.00 – 181.00	21.70 – 25.43
5	n	45	45	45	45
	M	29.84	70.93	174.07	23.32
	SI	7.57	12.04	8.61	2.90
	95% CI	27.57 – 32.12	67.31 – 74.55	171.48 – 176.65	22.45 – 24.19
	range	19.00 – 55.00	51.20 – 100.00	155.00 – 194.00	19.20 – 29.38
	Me	27.00	70.00	175.00	22.79
	Q1 – Q3	25.00 – 33.00	62.20 – 76.00	168.00 – 180.00	21.25 – 24.22
p between groups according to *Kruskal–Wallis one-way ANOVA*		0.590	0.036	0.107	0.264

Table S3: Volunteer demographic data overview for phase IIb.

Group	Variable	Age (full years)	Weight (kg)	Height (cm)	BMI (kg/m^2)
1	n	215	215	215	215
	M	41,83	72,86	171,02	24,82
	SI	14,21	10,47	8,39	2,33
	95% CI	39,92 – 43,74	71,45 – 74,27	169,89 – 172,15	24,50 – 25,13
	range	18,00 – 73,00	49,00 – 97,00	153,00 – 189,00	18,86 – 29,94
	Me	42,00	73,20	172,00	24,68
	Q1 – Q3	30,00 – 53,00	65,15 – 79,80	164,00 – 178,00	23,09 – 26,58
2	n	218	218	218	218
	M	43,52	72,02	171,07	24,54
	SI	14,92	10,45	8,37	2,50
	95% CI	41,53 – 45,51	70,63 – 73,42	169,95 – 172,18	24,21 – 24,87
	range	18,00 – 84,00	47,60 – 101,40	156,00 – 195,00	18,78 – 29,89
	Me	42,00	71,70	170,00	24,31
	Q1 – Q3	32,00 – 55,75	64,40 – 78,97	164,00 – 178,00	22,68 – 26,44
p between groups according to *Kruskal–Wallis one-way ANOVA*		0,279	0,302	0,937	0,231

Table S4: Number and % (in brackets) of volunteers experiencing at least one AE and total AE count (NOE = Number of Events) for groups 1 and 2 in phase I safety assessment, for each AE type or system affected. Medical Dictionary for Regulatory Activities (MedDRA) codes are provided for each category and AE.

MedDRA SOC	MedDRA PT	Group 1 (n = 5)	Group 2 (n = 15)
10005329 Blood and lymphatic system disorders	10024384 Leukopenia	1 (20.00%) NOE = 1	0 (0.00%) NOE = 0
10015919 Eye disorders	10013774 Dry eye	0 (0.00%) NOE = 0	1 (6.67%) NOE = 1
10017947 Digestive system disorders	10000081 Abdominal pain	1 (20.00%) NOE = 2	0 (0.00%) NOE = 0
	10012735 Diarrhoea	0 (0.00%) NOE = 0	1 (6.67%) NOE = 1
	10047700 Vomiting	0 (0.00%) NOE = 0	1 (6.67%) NOE = 1
10018065 General disorders and administration site conditions	10008531 Chills	0 (0.00%) NOE = 0	1 (6.67%) NOE = 1
	10016256 Fatigue	1 (20.00%) NOE = 3	3 (20.00%) NOE = 3
	10022061 Injection site erythema	0 (0.00%) NOE = 0	1 (6.67%) NOE = 1
	10022075 Injection site induration	0 (0.00%) NOE = 0	1 (6.67%) NOE = 1
	10022086 Injection site pain	3 (60.00%) NOE = 3	7 (46.67%) NOE = 7

MedDRA SOC	MedDRA PT	Group 1 (n = 5)	Group 2 (n = 15)
	10022093 Injection site pruritus	0 (0.00%) NOE = 0	1 (6.67%) NOE = 1
	10025482 Malaise	1 (20.00%) NOE = 3	3 (20.00%) NOE = 3
	10037660 Pyrexia	0 (0.00%) NOE = 0	1 (6.67%) NOE = 1
10022891 Investigations	10003481 Aspartate aminotransferase increased	0 (0.00%) NOE = 0	1 (6.67%) NOE = 1
	10005750 Blood pressure increased	1 (20.00%) NOE = 1	0 (0.00%) NOE = 0
	10005758 Blood pressure systolic decreased	1 (20.00%) NOE = 1	0 (0.00%) NOE = 0
	10005760 Blood pressure systolic increased	1 (20.00%) NOE = 1	0 (0.00%) NOE = 0
	10019301 Heart rate decreased	2 (40.00%) NOE = 2	0 (0.00%) NOE = 0
	10019303 Heart rate increased	1 (20.00%) NOE = 1	0 (0.00%) NOE = 0
	10025258 Lymphocyte count increased	1 (20.00%) NOE = 1	2 (13.33%) NOE = 2
	10029366 Neutrophil count decreased	1 (20.00%) NOE = 2	0 (0.00%) NOE = 0
	10047942 Leukocyte count decreased	1 (20.00%) NOE = 1	0 (0.00%) NOE = 0
	10047943 Leukocyte count increased	0 (0.00%) NOE = 0	1 (6.67%) NOE = 1
	10049187 Red blood cell sedimentation rate increased	0 (0.00%) NOE = 0	1 (6.67%) NOE = 1
10028395 Musculoskeletal and connective tissue disorders	10003239 Arthralgia	0 (0.00%) NOE = 0	1 (6.67%) NOE = 1
	10028411 Myalgia	1 (20.00%) NOE = 1	1 (6.67%) NOE = 1
10029205 Nervous system disorders	10019211 Headache	1 (20.00%) NOE = 3	3 (20.00%) NOE = 3
10037175 Psychiatric disorders	10010305 Confusion state	1 (20.00%) NOE = 1	0 (0.00%) NOE = 0
10038738 Respiratory. thoracic and mediastinal disorders	10011224 Cough	1 (20.00%) NOE = 1	0 (0.00%) NOE = 0
	10013968 Dyspnoea	0 (0.00%) NOE = 0	1 (6.67%) NOE = 1
Total		4 (80.00%) NOE = 28	9 (60.00%) NOE = 32

Table S5: Number and % (in brackets) of volunteers experiencing at least one AE and total AE count (NOE) for groups 3,4 and 5 in phase II safety assessment, for each AE type or system affected. Medical Dictionary for Regulatory Activities (MedDRA) codes are provided.

MedDRA SOC	MedDRA PT	Group 3, double vaccination (n = 44)	Group 4, single vaccination (n = 45)	Group 5, placebo (n = 45)
10013993 Ear and labyrinth disorders	10014020 Ear pain	1 (2.27%) NOE = 1	0 (0.00%) NOE = 0	0 (0,00%) NOE = 0
10015919 Eye disorders	10013774 Dry eye	2 (4.55%) NOE = 3	0 (0.00%) NOE = 0	0 (0.00%) NOE = 0
10017947 Gastrointestinal disorders	10000081 Abdominal pain	2 (4.55%) NOE = 2	2 (4.44%) NOE = 2	0 (0.00%) NOE = 0
	10012735 Diarrhoea	2 (4.55%) NOE = 3	1 (2.22%) NOE = 1	1 (2.22%) NOE = 1
	10047700 Vomiting	1 (2.27%) NOE = 1	0 (0.00%) NOE = 0	0 (0.00%) NOE = 0
10018065 General disorders and administration site conditions	10003549 Asthenia	0 (0.00%) NOE = 0	1 (2.22%) NOE = 1	1 (2.22%) NOE = 1
	10008531 Chills	2 (4.55%) NOE = 2	2 (4.44%) NOE = 2	0 (0.00%) NOE = 0
	10016256 Fatigue	2 (4.55%) NOE = 3	3 (6.67%) NOE = 3	1 (2.22%) NOE = 1
	10020843 Hyperthermia	2 (4.55%) NOE = 2	1 (2.22%) NOE = 1	0 (0.00%) NOE = 0
	10022004 Influenza like illness	3 (6.82%) NOE = 3	5 (11.11%) NOE = 5	5 (11.11%) NOE = 5
	10022061 Injection site erythema	4 (9.09%) NOE = 6	11 (24.44%) NOE = 12	1 (2.22%) NOE = 1
	10022075 Injection site induration	9 (20.45%) NOE = 13	14 (31.11%) NOE = 16	5 (11.11%) NOE = 5
	10022085 Injection site oedema	4 (9.09%) NOE = 5	6 (13.33%) NOE = 9	0 (0.00%) NOE = 0
	10022086 Injection site pain	34 (77.27%) NOE = 53	31 (68.89%) NOE = 43	12 (26.67%) NOE = 14
	10022093 Injection site pruritus	7 (15.91%) NOE = 8	5 (11.11%) NOE = 5	0 (0.00%) NOE = 0
	10025482 Malaise	4 (9.09%) NOE = 5	3 (6.67%) NOE = 4	2 (4.44%) NOE = 2
	10037660 Pyrexia	4 (9.09%) NOE = 5	1 (2.22%) NOE = 1	3 (6.67%) NOE = 4
	10061458 Feeling of body temperature change	0 (0.00%) NOE = 0	1 (2.22%) NOE = 1	0 (0.00%) NOE = 0
	10075107 Haemodynamic oedema	0 (0.00%) NOE = 0	1 (2.22%) NOE = 1	0 (0.00%) NOE = 0
10021881 Infections and infestations	10019973 Herpesvirus infection	0 (0.00%) NOE = 0	0 (0.00%) NOE = 0	1 (2.22%) NOE = 1

MedDRA SOC	MedDRA PT	Group 3, double vaccination (n = 44)	Group 4, single vaccination (n = 45)	Group 5, placebo (n = 45)
	10044302 Tracheitis	0 (0.00%) NOE = 0	1 (2.22%) NOE = 1	1 (2.22%) NOE = 1
	10062106 Infective pneumonia	1 (2.27%) NOE = 1	0 (0.00%) NOE = 0	1 (2.22%) NOE = 1
	10067152 Oral herpes	0 (0.00%) NOE = 0	0 (0.00%) NOE = 0	1 (2.22%) NOE = 1
	10084268 COVID-19	3 (6.82%) NOE = 3	3 (6.67%) NOE = 3	5 (11.11%) NOE = 5
10022117 Injury. poisoning and procedural complications	10016970 Foot fracture	1 (2.27%) NOE = 1	0 (0.00%) NOE = 0	0 (0.00%) NOE = 0
10028395 Musculoskeletal and connective tissue disorders	10003239 Arthralgia	2 (4.55%) NOE = 3	0 (0.00%) NOE = 0	1 (2.22%) NOE = 1
	10003988 Back pain	0 (0.00%) NOE = 0	0 (0.00%) NOE = 0	1 (2.22%) NOE = 1
	10028411 Myalgia	4 (9.09%) NOE = 4	2 (4.44%) NOE = 3	0 (0.00%) NOE = 0
10029205 Nervous system disorders	10019211 Headache	6 (13.64%) NOE = 14	4 (8.89%) NOE = 4	3 (6.67%) NOE = 5
10037175 Psychiatric disorders	10010305 Confusion state	0 (0.00%) NOE = 0	1 (2.22%) NOE = 1	0 (0.00%) NOE = 0
10038738 Respiratory. thoracic and mediastinal disorders	10011224 Cough	1 (2.27%) NOE = 2	2 (4.44%) NOE = 2	2 (4.44%) NOE = 2
	10043521 Throat irritation	1 (2.27%) NOE = 1	1 (2.22%) NOE = 1	1 (2.22%) NOE = 1
10040785 Skin and subcutaneous tissue disorders	10020642 Hyperhidrosis	2 (4.55%) NOE = 3	1 (2.22%) NOE = 1	0 (0.00%) NOE = 0
	10037844 Rash	0 (0.00%) NOE = 0	1 (2.22%) NOE = 1	0 (0.00%) NOE = 0
Total		37 (84.09%) NOE = 147	37 (82.22%) NOE = 124	23 (51.11%) NOE = 53

Table S6: Number and % (in brackets) of volunteers experiencing at least one AE and total AE count (NOE) for groups 1 and 2 in phase IIb safety assessment, for each AE type or system affected. Medical Dictionary for Regulatory Activities (MedDRA) codes are provided.

MedDRA SOC	MedDRA PT	Group 1, single vaccination (n = 215)	Group 2, double vaccination (n = 218)
10005329 Blood and lymphatic system disorders	10025197 Lymphadenopathy	0 (0.00%) NOE = 0	3 (1.38%) NOE = 6
10013993 Ear and labyrinth disorders	10014020 Ear pain	0 (0.00%) NOE = 0	1 (0.46%) NOE = 1
10015919 Eye disorders	10003552 Asthenopia	0 (0.00%) NOE = 0	1 (0.46%) NOE = 1
	10034960 Photophobia	0 (0.00%) NOE = 0	1 (0.46%) NOE = 1
10017947 Gastrointestinal disorders	10000081 Abdominal pain	2 (0.93%) NOE = 2	4 (1.83%) NOE = 5

MedDRA SOC	MedDRA PT	Group 1, single vaccination (n = 215)	Group 2, double vaccination (n = 218)
	10000087 Abdominal pain upper	2 (0.93%) NOE = 2	0 (0.00%) NOE = 0
	10012735 Diarrhoea	7 (3.26%) NOE = 9	3 (1.38%) NOE = 3
	10028813 Nausea	3 (1.40%) NOE = 3	2 (0.92%) NOE = 2
	10044055 Toothache	0 (0.00%) NOE = 0	1 (0.46%) NOE = 1
10018065 General disorders and administration site conditions	10003549 Asthenia	5 (2.33%) NOE = 5	7 (3.21%) NOE = 7
	10008531 Chills	11 (5.12%) NOE = 12	9 (4.13%) NOE = 10
	10016256 Fatigue	0 (0.00%) NOE = 0	1 (0.46%) NOE = 1
	10016334 Feeling hot	1 (0.47%) NOE = 1	0 (0.00%) NOE = 0
	10022061 Injection site erythema	24 (11.16%) NOE = 28	24 (11.01%) NOE = 26
	10022066 Injection site hematoma	1 (0.47%) NOE = 1	0 (0.00%) NOE = 0
	10022075 Injection site induration	17 (7.91%) NOE = 18	26 (11.93%) NOE = 28
	10022085 Injection site oedema	10 (4.65%) NOE = 11	15 (6.88%) NOE = 15
	10022086 Injection site pain	71 (33.02%) NOE = 82	77 (35.32%) NOE = 94
	10022093 Injection site pruritus	15 (6.98%) NOE = 15	14 (6.42%) NOE = 16
	10025482 Malaise	14 (6.51%) NOE = 15	17 (7.80%) NOE = 17
	10033371 Pain	3 (1.40%) NOE = 3	8 (3.67%) NOE = 9
	10037660 Pyrexia	8 (3.72%) NOE = 9	7 (3.21%) NOE = 8
	10043458 Thirst	1 (0.47%) NOE = 1	0 (0.00%) NOE = 0
	10049438 General physical health deterioration	0 (0.00%) NOE = 0	2 (0.92%) NOE = 2
	10054266 Injection site discomfort	0 (0.00%) NOE = 0	1 (0.46%) NOE = 1
	10068879 Vaccination site tenderness	1 (0.47%) NOE = 1	0 (0.00%) NOE = 0
10021881 Infections and infestations	10011781 Cystitis	0 (0.00%) NOE = 0	1 (0.46%) NOE = 1
	10014011 Ear infection	0 (0.00%) NOE = 0	1 (0.46%) NOE = 1
	10039083 Rhinitis	0 (0.00%) NOE = 0	1 (0.46%) NOE = 1
	10044008 Tonsillitis	0 (0.00%) NOE = 0	1 (0.46%) NOE = 1
	10046571 Urinary tract infection	0 (0.00%) NOE = 0	1 (0.46%) NOE = 1
	10062106 Respiratory tract viral infection	5 (2.33%) NOE = 5	2 (0.92%) NOE = 2
	10084268 COVID-19	0 (0.00%) NOE = 0	1 (0.46%) NOE = 1
10022117 Injury. poisoning and procedural complications	10002544 Ankle fracture	0 (0.00%) NOE = 0	1 (0.46%) NOE = 1
	10050584 Contusion	1 (0.47%) NOE = 1	0 (0.00%) NOE = 0
10022891 Investigations	10005750 Blood pressure increase	1 (0.47%) NOE = 1	1 (0.46%) NOE = 1
10027433 Metabolism and nutrition disorders	10061428 Decreased appetite	2 (0.93%) NOE = 2	5 (2.29%) NOE = 5
10028395 Musculoskeletal and connective tissue disorders	10003239 Arthralgia	2 (0.93%) NOE = 3	7 (3.21%) NOE = 8
	10003988 Back pain	1 (0.47%) NOE = 1	2 (0.92%) NOE = 2

MedDRA SOC	MedDRA PT	Group 1, single vaccination (n = 215)	Group 2, double vaccination (n = 218)
	10028334 Muscle spasms	0 (0.00%) NOE = 0	2 (0.92%) NOE = 2
	10028372 Muscular weakness	7 (3.26%) NOE = 8	12 (5.50%) NOE = 12
	10028411 Myalgia	4 (1.86%) NOE = 4	9 (4.13%) NOE = 10
10029205 Nervous system disorders	10013573 Dizziness	6 (2.79%) NOE = 6	14 (6.42%) NOE = 15
	10019211 Headache	25 (11.63%) NOE = 25	36 (16.51%) NOE = 42
	10041349 Somnolence	1 (0.47%) NOE = 1	1 (0.46%) NOE = 1
10037175 Psychiatric disorders	10022437 Insomnia	8 (3.72%) NOE = 8	9 (4.13%) NOE = 9
	10024642 Listlessness	0 (0.00%) NOE = 0	1 (0.46%) NOE = 1
10038359 Renal and urinary disorders	10013990 Dysuria	0 (0.00%) NOE = 0	1 (0.46%) NOE = 1
10038738 Respiratory. thoracic and mediastinal disorders	10011224 Cough	1 (0.47%) NOE = 1	4 (1.83%) NOE = 4
	10028735 Nasal congestion	0 (0.00%) NOE = 0	3 (1.38%) NOE = 4
	10039101 Rhinorrhea	1 (0.47%) NOE = 1	4 (1.83%) NOE = 4
	10068319 Oropharyngeal pain	6 (2.79%) NOE = 6	5 (2.29%) NOE = 5
	10080125 Throat clearing	1 (0.47%) NOE = 1	0 (0.00%) NOE = 0
10040785 Skin and subcutaneous tissue disorders	10001760 Alopecia	0 (0.00%) NOE = 0	1 (0.46%) NOE = 1
	10020642 Hyperhidrosis	10 (4.65%) NOE = 12	8 (3.67%) NOE = 8
10047065 Vascular disorders	10020772 Hypertension	2 (0.93%) NOE = 2	0 (0.00%) NOE = 0
	10020802 Hypertensive crisis	2 (0.93%) NOE = 2	1 (0.46%) NOE = 1
Total		132 (61.40%) NOE = 309	133 (61.01%) NOE = 399

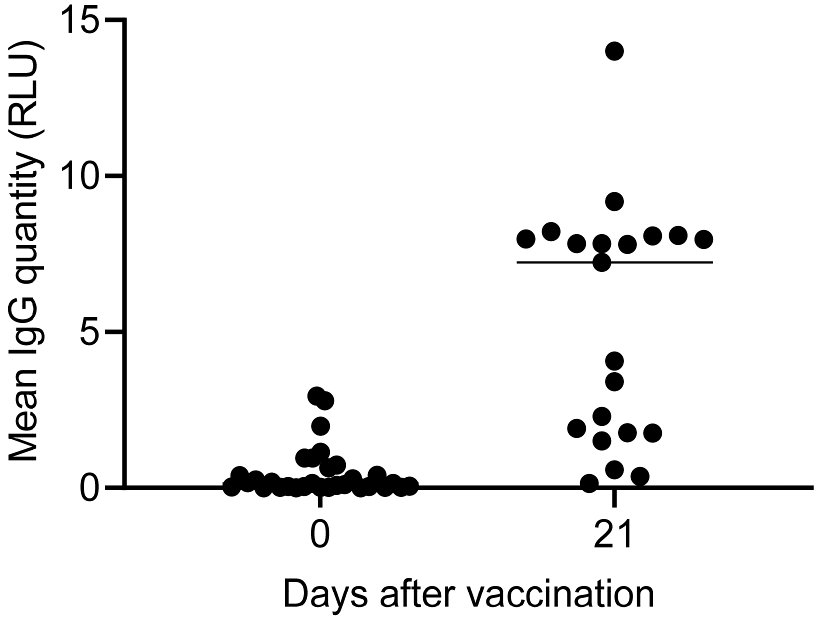

Figure S1: Anti-N IgG quantification in sera of volunteers of groups 1 and 2 (combined data) in phase I via a chemiluminescent immunoassay. Points represent individual values, bars represent the medians. RLU is relative light units used by the assay. N=20. p<0.0001 between 0 and 21 day values according to Mann-Whitney test.

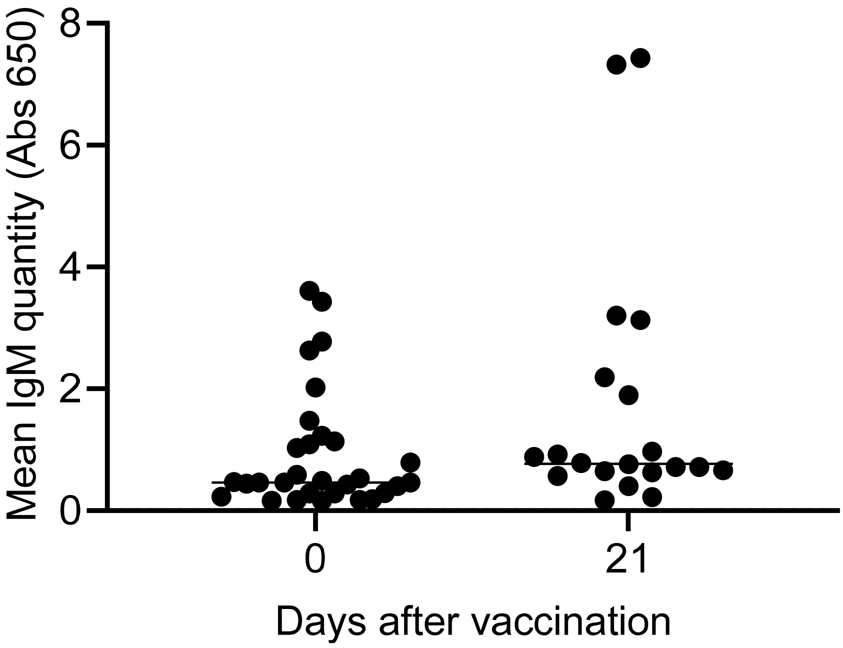

Figure S2: Anti-N IgM quantification in sera of volunteers of groups 1 and 2 (combined data) in phase I via ELISA. Points represent individual values, bars represent the medians. N=20. P=0.0615 between 0 and 21 day values according to Mann-Whitney test.

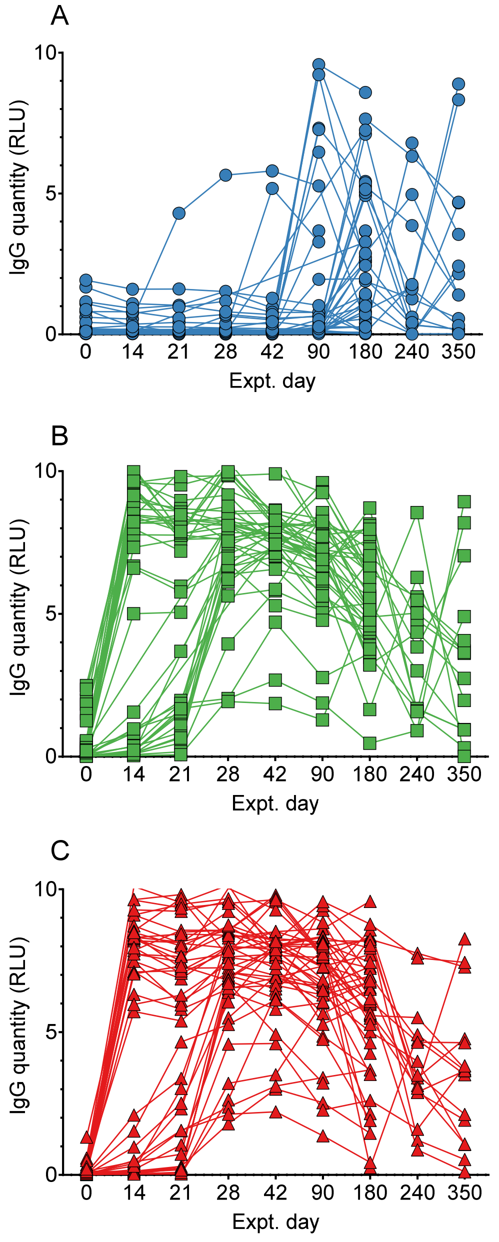

Figure S3: Individual values for anti-N IgG quantification via a chemiluminescent immunoassay in sera of volunteers of the placebo (A), single vaccination (B) and double vaccination (C) groups in phase II. N = 44 for each group, for days 0-180, for days 240-350, n = 15 for placebo and single vaccination groups and 16 for double vaccination group. Data points may be omitted due to either experimental errors or inability to procure or transport samples. RLU is relative light units used by the assay.

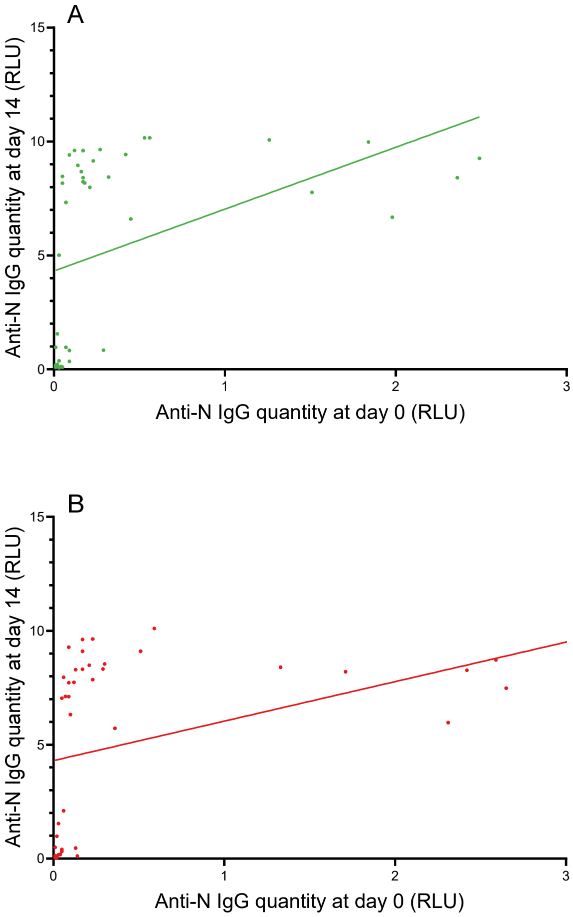

Figure S4: Values on day 0 vs. values on day 14 of anti-N IgG quantities in sera of volunteers vaccinated once (A) or twice (B) with Convacell® during phase II, as detected via a chemiluminescent immunoassay. RLU is relative light units used by the assay. P<0.05 of the slope of the fitted line being significantly non-zero for both groups, according to simple linear regression analysis.

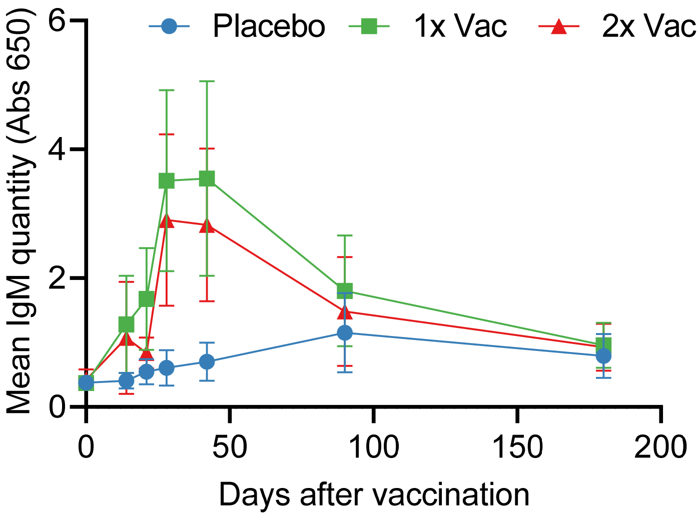

Figure S5: Anti-N IgM quantification in sera of volunteers of the placebo group, single vaccination group (1x Vac) and double vaccination group (2x Vac) in phase II via ELISA. Points represent median values; error bars represent 95% confidence intervals. N = 44 for placebo and single vaccination groups, n = 45 for double vaccination group.

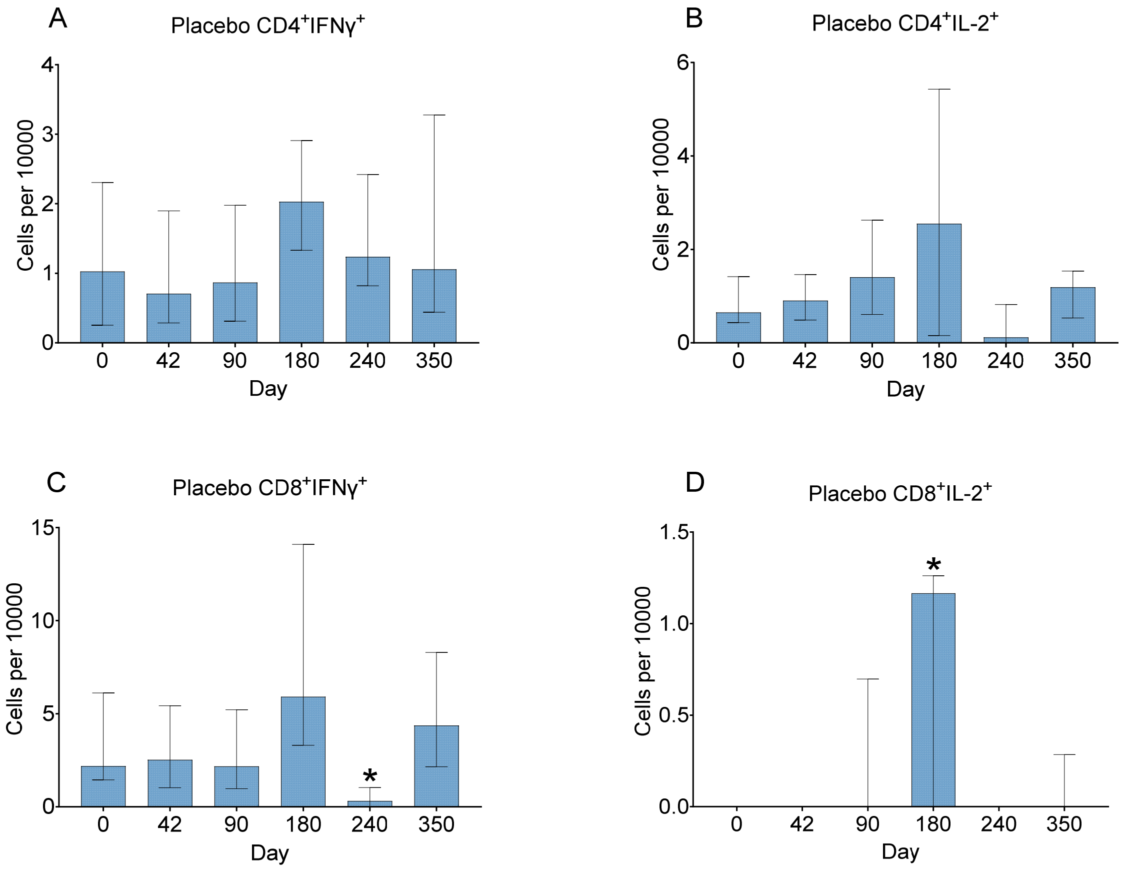

Figure S6: Phenotype abundances of T-lymphocytes stimulated in response to treatment with N protein in the placebo-injected group by day after first vaccination for A) $CD4^{+}IFN\gamma^{+}$ T lymphocytes, B) $CD4^{+}IL\text{-}2^{+}$ T lymphocytes, C) $CD8^{+}IFN\gamma^{+}$ T lymphocytes and D) $CD8^{+}IL\text{-}2^{+}$ T lymphocytes. Points represent median values; error bars represent interquartile range. Asterisks represent statistically significant difference ($P<0.05$) between the indicated value and that at day 0, according to Wilcoxon signed-rank test.

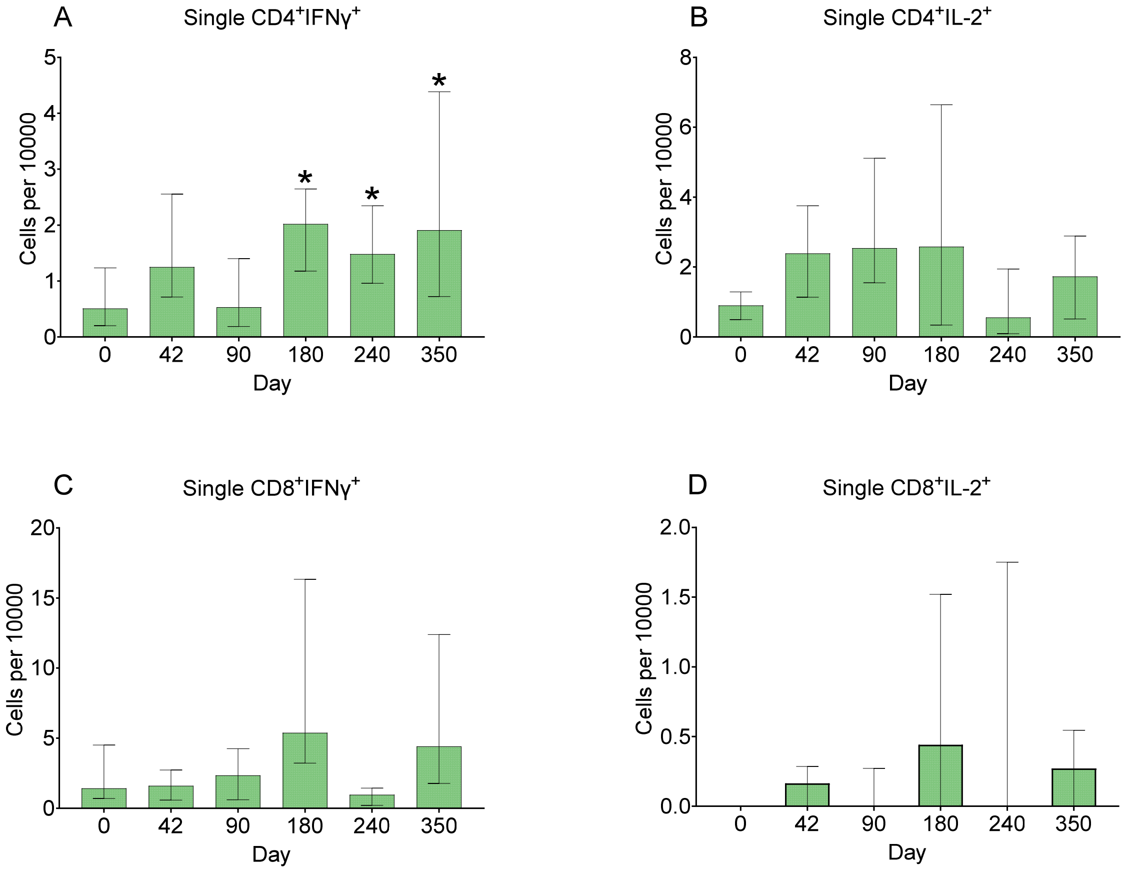

Figure S7: Phenotype abundances of T-lymphocytes stimulated in response to treatment with N protein in the single vaccination group by day after first vaccination for A) $CD4^{+}IFN\gamma^{+}$ lymphocytes, B) $CD4^{+}IL\text{-}2^{+}$ lymphocytes, C) $CD8^{+}IFN\gamma^{+}$ lymphocytes and D) $CD8^{+}IL\text{-}2^{+}$ lymphocytes. Points represent median values; error bars represent interquartile range. Asterisks represent statistically significant difference ($P<0.05$) between the indicated value and that at day 0, according to Wilcoxon signed-rank test.

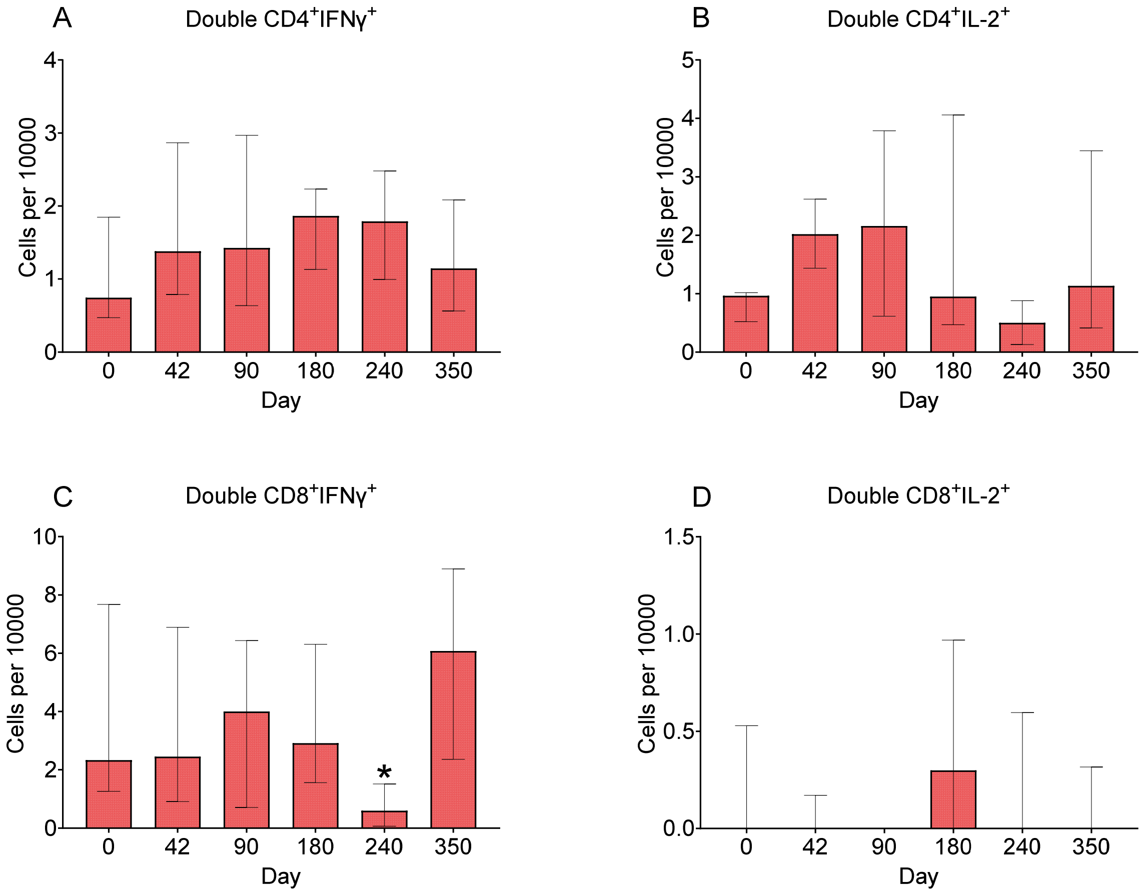

Figure S8: Phenotype abundances of T-lymphocytes stimulated in response to treatment with N protein in the single vaccination group by day after first vaccination for A) $CD4^{+}IFN\gamma^{+}$ lymphocytes, B) $CD4^{+}IL\text{-}2^{+}$ lymphocytes, C) $CD8^{+}IFN\gamma^{+}$ lymphocytes and D) $CD8^{+}IL\text{-}2^{+}$ lymphocytes. Points represent median values; error bars represent interquartile range. Asterisks represent statistically significant difference ($P<0.05$) between the indicated value and that at day 0, according to Wilcoxon signed-rank test.

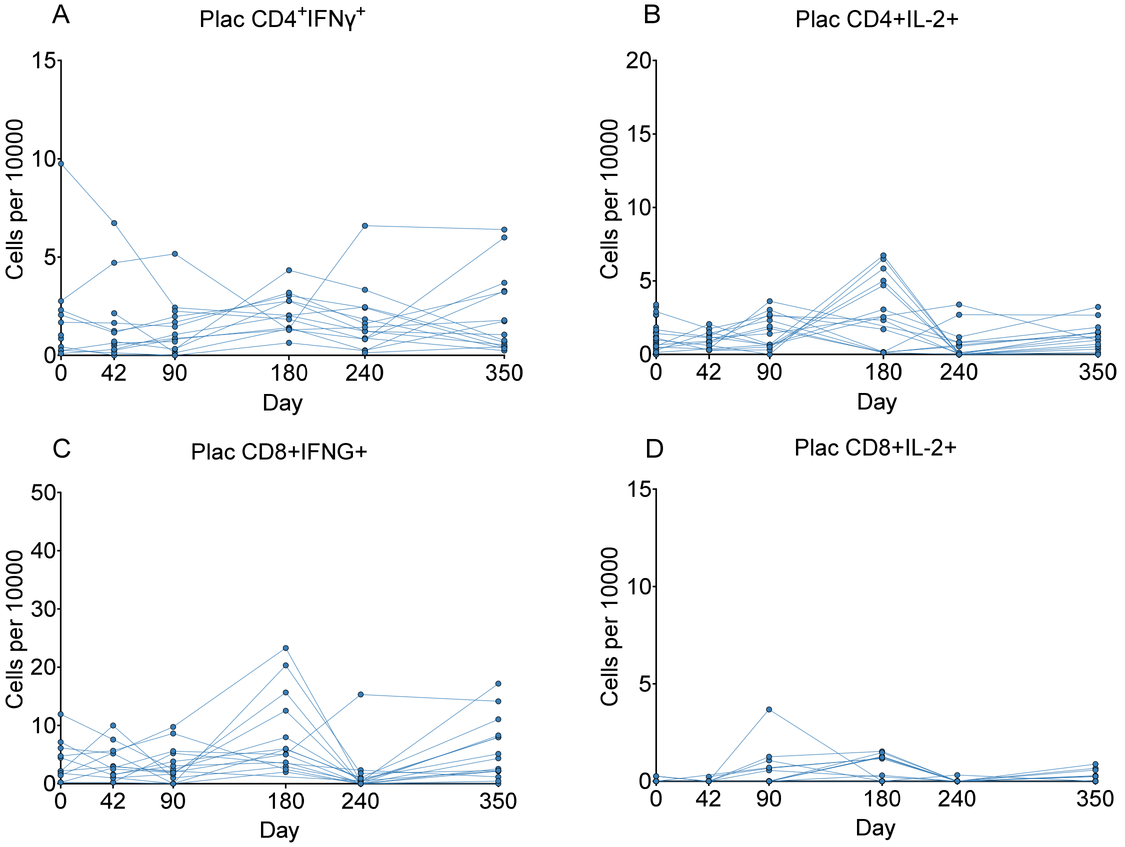

Figure S9: Individual values for phenotype abundances of T-lymphocytes stimulated in response to treatment with N protein in the placebo group by day after first vaccination for A) $CD4^{+}IFN\gamma^{+}$ lymphocytes, B) $CD4^{+}IL\text{-}2^{+}$ lymphocytes, C) $CD8^{+}IFN\gamma^{+}$ lymphocytes and D) $CD8^{+}IL\text{-}2^{+}$ lymphocytes.

`

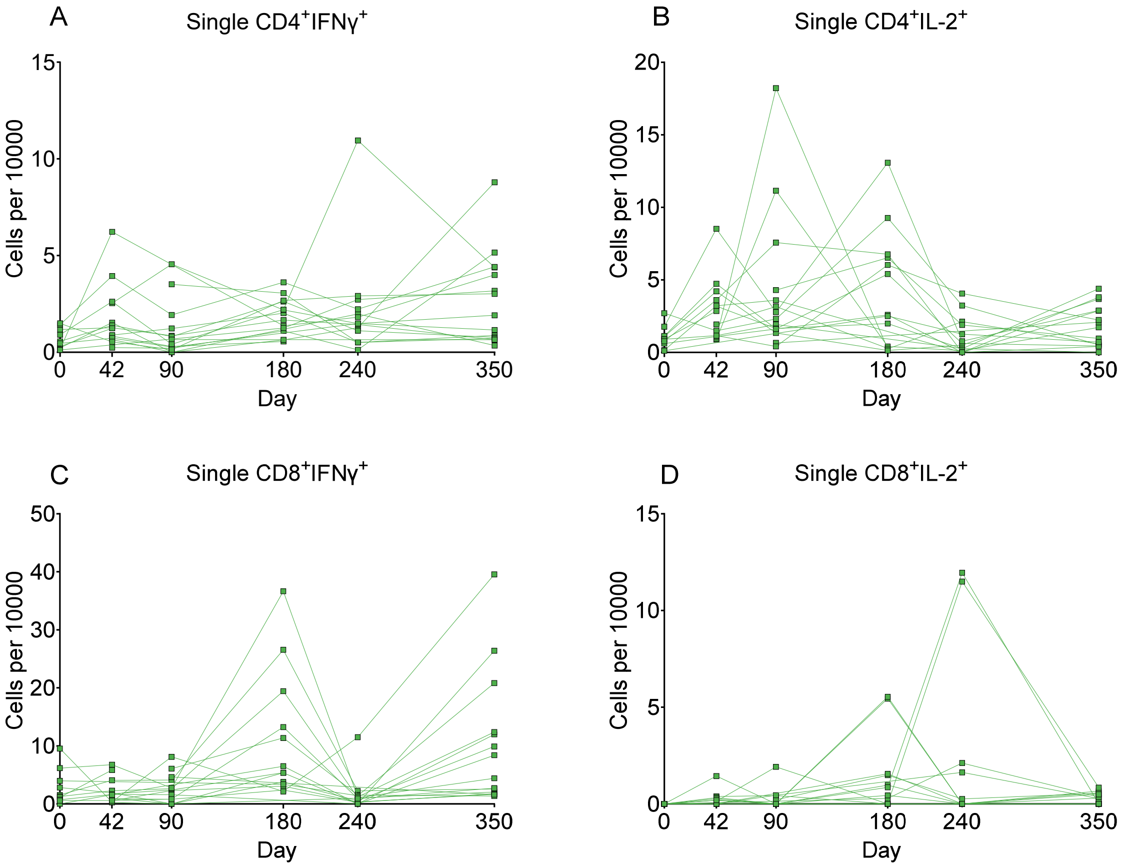

Figure S10: Individual values for phenotype abundances of T-lymphocytes stimulated in response to treatment with N protein in the single vaccination group by day after first vaccination for A) $CD4^{+}IFN\gamma^{+}$ lymphocytes, B) $CD4^{+}IL\text{-}2^{+}$ lymphocytes, C) $CD8^{+}IFN\gamma^{+}$ lymphocytes and D) $CD8^{+}IL\text{-}2^{+}$ lymphocytes.

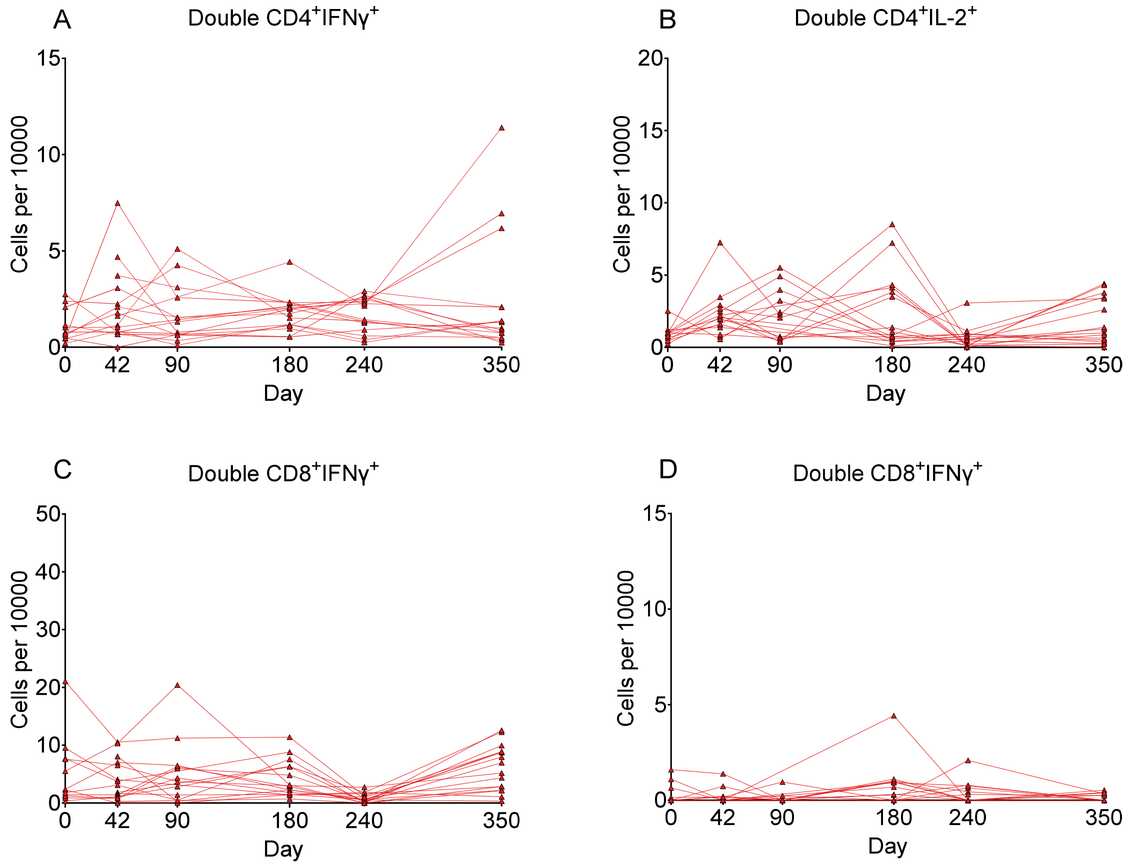

Figure S11: Individual values for phenotype abundances of T-lymphocytes stimulated in response to treatment with N protein in the double vaccination group by day after first vaccination for A) $CD4^{+}IFN\gamma^{+}$ lymphocytes, B) $CD4^{+}IL\text{-}2^{+}$ lymphocytes, C) $CD8^{+}IFN\gamma^{+}$ lymphocytes and D) $CD8^{+}IL\text{-}2^{+}$ lymphocytes.

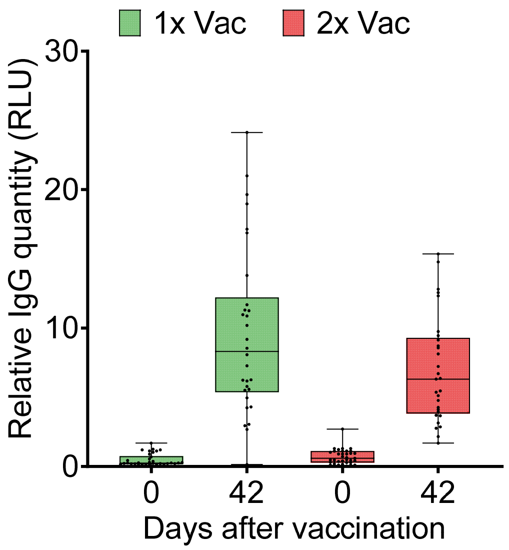

Figure S12: Specific anti-N IgG quantification by a chemiluminescent immunoassay in the sera of volunteers aged 60+ who received either one (1x Vac) or two (2x Vac) doses of Convacell®, on experiment days 0 and 42. Boxes represent interquartile range, lines are at medians. RLU is relative light units used by the assay. Values above 50 RFU are not plotted for reasons of scale. According to Wilcoxon's signed-rank test, $p<0.0001$ between day 0 and 42 values in each group. Between groups, according to Mann-Whitney test, $p=0.035$ on day 0, but $p= 0.094$ on day 42.

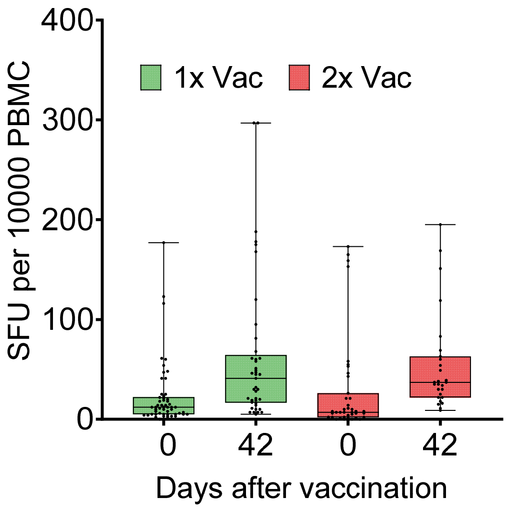

Figure S13: N-specific IFNγ^{+} PBMC quantification via ELISPOT in the blood of volunteers aged 60+ who received either one (1x Vac) or two (2x Vac) doses of Convacell®, on experiment days 0 and 42. Boxes represent interquartile range, lines are at medians. SFU is spot-forming units used by the assay, PMBC is peripheral blood monocytes. According to Wilcoxon's signed-rank test, $p<0.0001$ between day 0 and 42 values in each group. Between groups, according to Mann-Whitney test, p=0.23 on day 0 and p=0.68 on day 42.

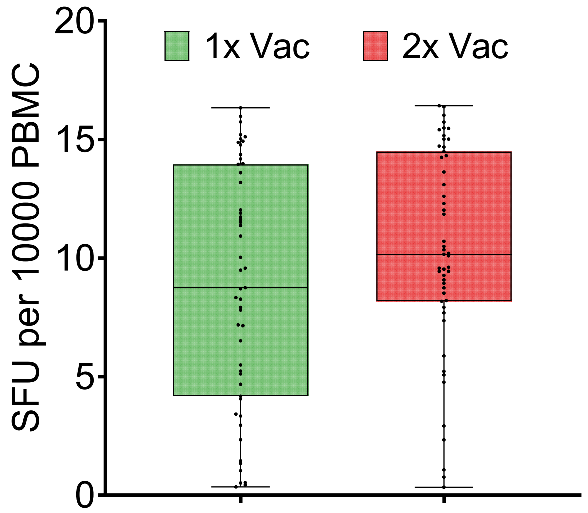

Figure S14: S-specific IFNγ+ PBMC quantification via ELISPOT in the blood of volunteers who received either one (1x Vac) or two (2x Vac) doses of Convacell®, on experiment day 0. Boxes represent interquartile range, lines are at medians. SFU is spot-forming units used by the assay, PMBC is peripheral blood monocytes. According to Mann-Whitney test, p= 0.13 between groups.

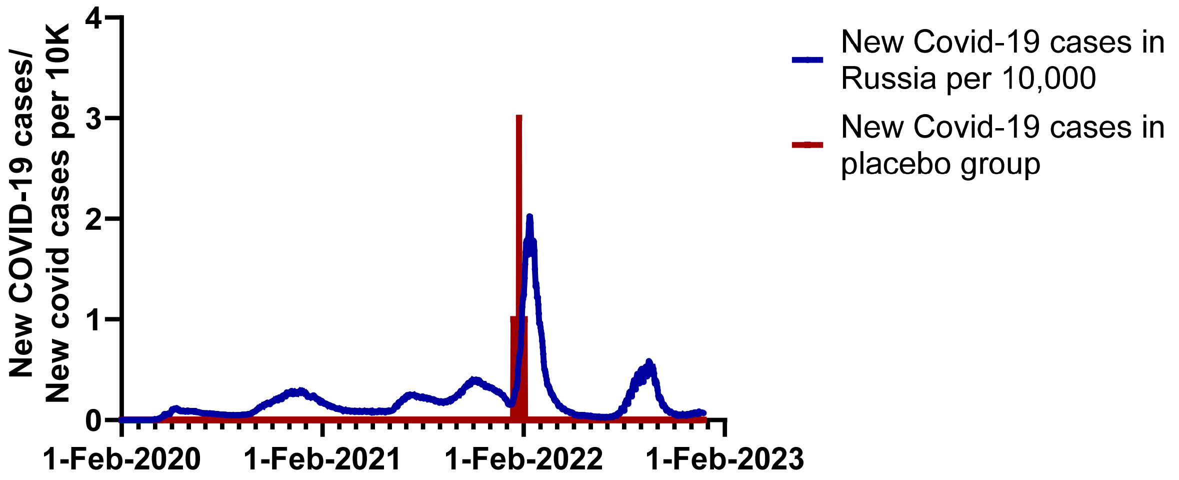

Figure S15: New COVID-19 cases in placebo group vs. new COVID-19 cases pet 10K in the general population in Russia in Feb. 2020 – Feb 2023. Data obtained from Statista: https://www.statista.com/statistics/1102303/coronavirus-new-cases-development-russia/. Accessed January 2023.
